# Evaluation of right ventriculoarterial coupling in pulmonary hypertension: a magnetic resonance study

**DOI:** 10.1186/1532-429X-13-S1-O73

**Published:** 2011-02-02

**Authors:** Javier Sanz, Ana Garcia-Alvarez, Leticia Fernandez-Friera, Ajith Nair, Jesus G Mirelis, Simonette Sawit, Sean Pinney, Valentin Fuster

**Affiliations:** 1 Mount Sinai School of Medicine, New York, NY, USA

## Introduction

Inadequate right ventriculo-arterial coupling is an important determinant of heart failure in pulmonary hypertension, in turn the main determinant of outcome in this disease. Coupling can be quantified as the ratio of pulmonary artery effective elastance (E_a_, an index of arterial load) to right ventricular maximal end-systolic elastance (E_max_, an index of contractility).

## Objective

To quantify right ventriculo-arterial coupling in pulmonary hypertension combining standard right heart catheterization and cardiac magnetic resonance (CMR), and to noninvasively estimate it with CMR alone.

## Methods

We included 139 patients undergoing CMR and right heart catheterization within 2 days (n=151 test pairs) for the evaluation of known or suspected pulmonary hypertension. Right ventricular end-systolic volume index (ESVI) and stroke volume index (SVI) were obtained, respectively, from cardiac cine images and phase-contrast of the pulmonary artery after adjusting for body surface area. Right heart catheterization provided mean pulmonary artery pressure (mPAP) as a surrogate of right ventricular end-systolic pressure, pulmonary capillary wedge pressure (PCWP), and pulmonary vascular resistance index (PVRI). E_a_ was calculated as (mPAP-PCWP)/SVI; and E_max_ as PAP/ESVI.

## Results

E_a_ increased linearly with advancing severity (as determined by PVRI quartiles; Figure, 1A), whereas E_max_ increased initially but tended to decrease subsequently (Figure, 1B). Thus, the ratio E_a_/E_max_ was maintained in earlier stages but increased markedly (indicating uncoupling) with more severe pulmonary hypertension (Figure, 1C). According to underlying etiologies and after adjustment for age, gender and PVRI, there were no significant differences amongst World Health Organization groups in terms of E_a_/E_max_. E_max_ was independently associated with right atrial pressure after adjustment for PVRI (β=-2.81, p<0.05). E_a_/E_max_ approximated noninvasively with CMR as ESVI/SVI equaled 0.75, 1.17, 2.28, and 3.51, for PVRI quartile groups (Q1 to Q4) respectively, showing excellent correlation with E_a_/E_max_ derived from invasive measurements (r=0.93, p<0.001) and progressing similarly with disease severity (p<0.001).

**Figure 1:**
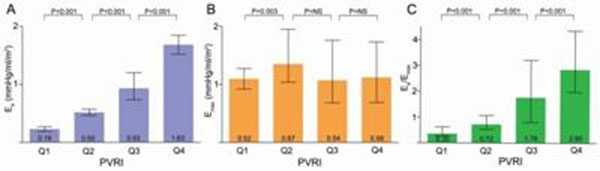
E_a_, E_max_ and E_a_/E_max_ according to pulmonary hypertension severity

## Conclusions

Right ventriculo-arterial coupling in pulmonary hypertension can be studied combining standard right heart catheterization and CMR indices. In addition, it can be approximated with CMR alone in a completely noninvasive fashion. Arterial load increases with disease severity whereas contractility cannot progress in parallel, leading to severe uncoupling.

